# Navigating Severe Thrombocytopenia in End-Stage Liver Disease: A Pre-transplant Management Dilemma

**DOI:** 10.7759/cureus.85811

**Published:** 2025-06-11

**Authors:** Fares Jamal, Amani Elshaer, Cody Eslinger, Sailendra Naidu, Rolland C Dickson, Leslie J Padrnos

**Affiliations:** 1 Hematology and Oncology, Mayo Clinic, Phoenix, USA; 2 Internal Medicine, Mayo Clinic, Phoenix, USA; 3 Interventional Radiology, Mayo Clinic, Phoenix, USA; 4 Gastroenterology and Hepatology, Mayo Clinic, Phoenix, USA

**Keywords:** end-stage liver disease (esld), immune thrombocytopenia purpura (itp), liver transplantation, partial splenic artery embolization (psae), refractory thrombocytopenia

## Abstract

End-stage liver disease (ESLD) due to autoimmune causes may be complicated by immune thrombocytopenia (ITP), making liver transplantation challenging. We present a case of refractory ITP in a liver transplant candidate and outline therapeutic strategies used to manage critical thrombocytopenia. A 60-year-old woman with ESLD from primary biliary cholangitis and autoimmune hepatitis was evaluated for liver and kidney transplants. Her platelet count declined from 45 K/µL to 12 K/µL, prompting hematology involvement. Despite intravenous immunoglobulin (IVIG) and dexamethasone, the platelet response was limited. Rituximab had no effect, and splenectomy was too risky pre-transplant due to portal hypertension. Thrombopoietin receptor agonists were limited by thrombotic risk. Subdural hematomas further complicated her course. Ultimately, partial splenic artery embolization improved her platelet count to 25 K/µL, allowing combined liver-kidney transplantation with splenectomy. Postoperatively, her platelet count increased to 854 K/µL without complications. Managing refractory ITP in ESLD requires a multifaceted approach. While corticosteroids and IVIG are first-line therapies, options like thrombopoietin agonists pose thrombotic risks. Splenic artery embolization may be a viable strategy to increase platelet counts before transplantation. Further research is needed to guide treatment in this complex population.

## Introduction

Thrombocytopenia is the most common hematologic abnormality in chronic liver disease, with a prevalence of up to 76% [1,2]. Its pathogenesis is multifactorial, with portal hypertension being a significant factor resulting in platelet sequestration in the spleen [3]. Other contributing factors include decreased platelet production due to inadequate thrombopoietin (TPO) synthesis and direct bone marrow suppression, especially in the context of viral or alcoholic hepatitis [1,4].

When thrombocytopenia is markedly severe in contrast to end-stage liver disease (ESLD), it's essential to investigate alternative causes such as immune thrombocytopenic purpura (ITP) [3]. In patients with ESLD, B lymphocytes overproduce glycoprotein (GP) IIb/IIIa, potentially increasing the risk of developing ITP [5]. This presents a significant bleeding risk, particularly in the peri-transplant period, where achieving target platelet thresholds may be more challenging [6]. Management typically includes corticosteroids and intravenous immunoglobulin (IVIG) [7].

This case highlights the challenge of managing severe thrombocytopenia in an ESLD, especially when liver transplantation is being considered. Despite facing resistance to several treatment lines, reaching a safe platelet threshold was essential before proceeding with transplantation. This highlights the difficulty of managing bleeding and thrombotic risks in complicated clinical situations, providing important insights for future management strategies.

## Case presentation

**Figure 1:**
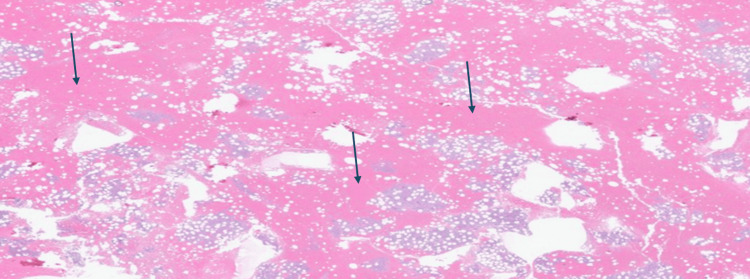
Bone marrow biopsy showing normocellular marrow without malignancy Arrows point to the normocellular cells in the bone marrow.

Our patient is a 60-year-old woman with a history of ESLD secondary to primary biliary cholangitis and autoimmune hepatitis, for which she was taking cholestyramine 4 mg daily and ursodiol 500 mg twice daily. She was referred to our facility for liver and kidney transplant evaluation. Prior to presentation, she had been recently diagnosed with ITP and treated with corticosteroids and IVIG, which resulted in only a transient improvement in her platelet count. At the time of presentation, her platelet count was 45 K/μL (reference range: 157-371 × 10⁹/L), subsequently declining to 12 K/μL, prompting hospital admission. Hematology and transplant teams were consulted. She was accepted for dual organ transplantation, requiring a platelet count above 25 K/μL to ensure a safe transplant. Per hematology’s evaluation, her thrombocytopenia was believed to be multifactorial, related to liver disease, splenic sequestration, and suspected ITP. For further evaluation, she underwent a bone marrow biopsy, which revealed normocellular marrow with trilineage hematopoiesis, occasional dysplastic megakaryocytes, and pancytopenia without evidence of malignancy (Figure 1).

Flow cytometry showed no immunophenotypic evidence of a monotypic B-cell or aberrant T-cell population.

A platelet threshold of 25K/μL was determined necessary for transplant surgery. She was started on intravenous immunoglobulin (IVIG) at 0.4 g/kg and dexamethasone 40 mg daily for four days, which led to a modest increase in platelet count from 12 K/μL to 20 K/μL. Given the limited response, multidisciplinary discussions were completed regarding second-line therapy options. Thrombopoietin receptor agonists (TPO-RA) were considered but avoided due to concerns for pre or post-transplant thrombosis. Splenectomy was considered but deferred due to the high operative risk in the setting of portal hypertension; it was reserved as an option to be performed at the time of transplant if platelet levels could be optimized. Rituximab was initiated as second-line therapy, but her platelet counts continued to drop despite two doses of treatment.

**Figure 2:**
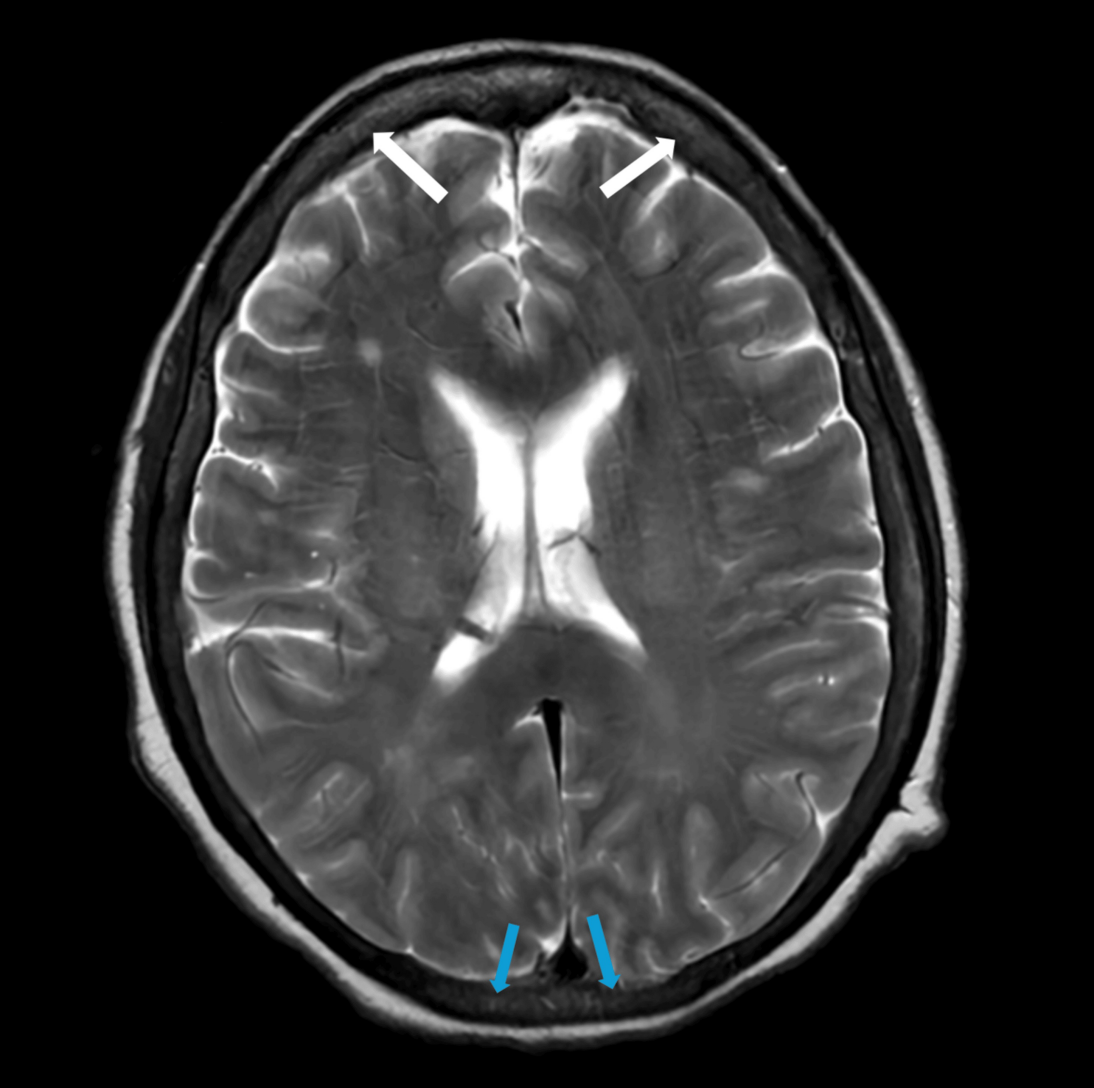
MRI brain imaging showing bilateral frontal and posterior fossa subdural hematomas White arrows point to the bilateral frontal subdural hematomas. Blue arrows point to the bilateral posterior subdural hematomas.

She remained dependent on human leukocyte antigen (HLA)-matched platelet transfusions, receiving a total of 7 units. Her hospitalization course was also complicated by new-onset persistent headaches. Brain imaging revealed new subdural hematomas in the bilateral frontal convexities and posterior fossa (Figure 2).

**Figure 3:**
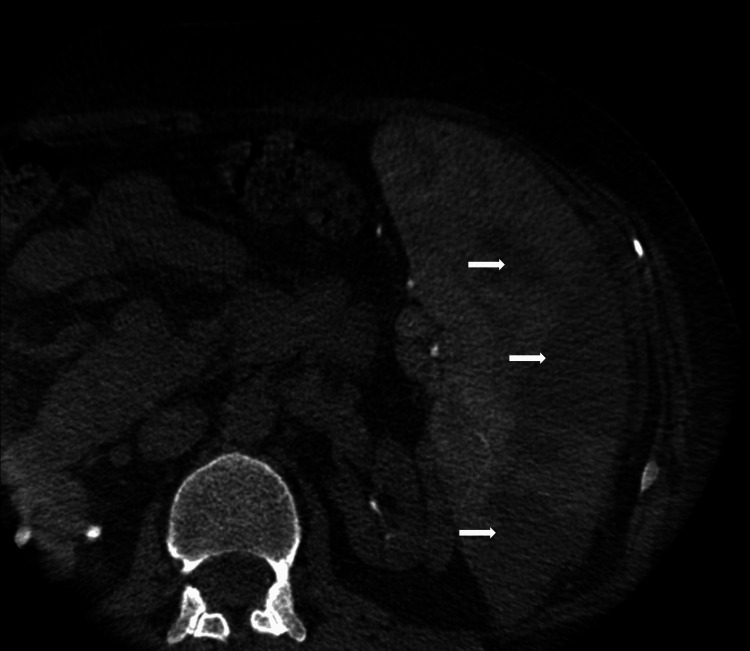
Imaging after partial splenic artery embolization (PSAE) using gel foam Arrows point to the areas affected by partial splenic artery embolization.

An alternative strategy considered was partial splenic artery embolization (PSAE) to decrease splenic sequestration. This was performed uneventfully (Figure 3).

Three days post-embolization, her platelet count improved from 15 K/μL to 25 K/μL, allowing for successful liver and kidney transplantation with intraoperative splenectomy.

**Figure 4:**
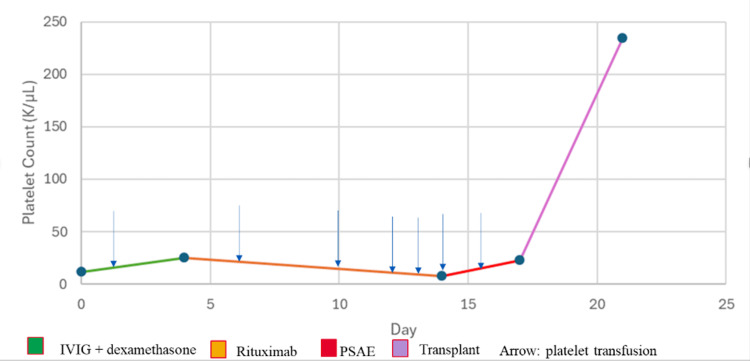
Platelet count over time with treatment interventions IVIG: intravenous immunoglobulin, PSAE: partial splenic artery embolization

Postoperatively, her platelet count rose to 75 K/μL on postoperative day (POD) 1, 174 K/μL by POD 5, and reached 854 K/μL approximately one month post-procedure (Figure 4).

Her postoperative course was uneventful with a favorable transplant outcome, and no graft rejection was observed. Immunosuppressive therapy was managed with 1 mg tacrolimus, 250 mg mycophenolate, and a four-month prednisone taper, with plans to continue low-dose prednisone indefinitely after completing the taper.

## Discussion

There are only a limited number of ITP cases reported in patients with ESLD within the literature [8,9]. Most of these cases have shown a positive response to corticosteroids and IVIG, underscoring their effectiveness as first-line treatments in this group. Our case reflects an intriguing trajectory of refractory ITP in the context of ESLD, which was not effectively addressed by conventional first-line therapies. This resulted in the postponement of transplantation and necessitated the optimization of advanced treatment strategies to alleviate these obstacles.

The presence of ITP in patients with ESLD undergoing liver transplantation presents a therapeutic challenge. The pathophysiology of thrombocytopenia in this setting is multifactorial, involving platelet sequestration in the spleen due to portal hypertension, impaired platelet production from bone marrow suppression, and inappropriately reduced TPO production [3]. Furthermore, in a study done by Satoh et al., B cells produced significantly more GPIIb/IIIa in ESLD patients compared to controls, potentially increasing the risk of ITP and contributing to severe thrombocytopenia [5].

Thrombocytopenia is a common barrier to performing high-risk procedures, including liver transplantation. Current guidelines recommend achieving a platelet threshold of at least 50,000/μL prior to procedures associated with a high risk of bleeding [6]. Platelet transfusion is the mainstay of management in such cases [10]. The management of thrombocytopenia in ESLD becomes more challenging when autoimmune diseases such as ITP contribute to the low platelet count. First-line of therapy includes corticosteroids, such as high-dose dexamethasone (40 mg daily for four days, up to three cycles) or prednisone/prednisolone (1 mg/kg/day for up to two weeks with tapering by six to eight weeks) [11]. With corticosteroid monotherapy, 60-80% of patients achieve a response.11 The addition of IVIG can increase the response rate to over 80% in newly diagnosed cases [12]. Second-line therapy includes rituximab, which produces a short response in 60-70% of patients; however, the onset of action might be delayed [13].

TPO-RAs have emerged as alternative treatment options, including romiplostim, eltrombopag, avatrombopag, and lusutrombopag [14,15]. However, their use in ESLD is associated with significant risks [16]. A study done by Yu et al. demonstrated that combining TPO-RA with dexamethasone results in a high response rate [17]. However, certain TPO-RAs, particularly eltrombopag, carry an increased risk of thromboembolic complications, including portal vein thrombosis, especially in patients with ESLD [16]. In a study by Loffredo et al., ESLD patients treated with eltrombopag had a higher incidence of thromboembolic events, including portal vein thrombosis, than controls [16]. These findings highlight the need for cautious use of TPO-RA in ESLD, particularly in patients with preexisting thrombotic risk factors or portal hypertension.

In refractory cases, laparoscopic splenectomy or PSAE may be considered [11]. However, laparoscopic splenectomy is associated with a higher risk of hemorrhage, sepsis, portal vein thrombosis, and mortality compared to PSAE [14]. A retrospective study demonstrated a significant platelet count increase in ESLD undergoing PSAE. Another systematic review and meta-analysis reported a 74.4% overall response rate in ITP patients treated with this approach [18,19]. Furthermore, PSAE can be performed from a radial approach decreasing the risk of bleeding complications in these profoundly thrombocytopenic patients [20]. These findings highlight PSAE as a promising treatment of thrombocytopenia in ESLD patients with ITP.

## Conclusions

This case highlights the challenges of managing severe thrombocytopenia in a patient with both ESLD and ITP, especially in the setting of organ transplantation. Despite multiple standard therapies, her platelet count remained critically low, putting her at high risk for life-threatening bleeding. PSAE was able to improve her platelet count such that she could undergo liver transplantation and splenectomy. This provided an effective solution when other treatments failed. Her successful recovery and transplant highlight the value of a multidisciplinary approach.
